# Critical interventions, diagnosis, and mortality in children treated by a physician-manned mobile emergency care unit

**DOI:** 10.1186/s13049-025-01346-x

**Published:** 2025-02-20

**Authors:** Alexandra Claire McKenzie, Mads Belger Risom, Jens-Jakob Kjer Møller, Johan Mikkelsen, Sarah Friis Skole-Sørensen, Vibe Maria Laden Nielsen, Nicola Groes Clausen, Søren Mikkelsen

**Affiliations:** 1 The Prehospital Research Unit, Region of Southern Denmark, Odense University Hospital, Odense, Denmark; 2 Department Anaesthesiology Intensive Care Medicine, Odense University Hospital, Odense, Denmark; 3 Open Patient Data Explorative Network, Odense University Hospital, Odense, Denmark; 4 Centre for Prehospital and Emergency Research, Aalborg University, Aalborg, Denmark; 5 Paediatric Anaesthesia Section, Department Anaesthesiology Intensive Care Medicine, Odense University Hospital, Odense, Denmark

**Keywords:** Prehospital critical care, Prehospital paediatrics, Critical prehospital interventions

## Abstract

**Background:**

The purpose of this study was to clarify the potentially life-saving critical interventions performed on children below the age of seven by the physician-manned mobile emergency care unit (MECU) in Odense, Denmark. We investigated critical interventions in relation to morbidity and mortality.

**Methods:**

A retrospective cohort study of all MECU missions involving children below the age of seven. The study period was from October 1 2007 to December 31 2020. Data sources were the MECU Odense database, the Danish National Patient Registry, and the Danish Civil Registration System. Variables were critical interventions, the severity of injury/illness, MECU on-scene time, in-hospital diagnosis and 7-day, 30-day, and 90-day mortality.

**Results:**

The MECU carried out 4,032 missions to children below 7 years. 88 patients (2.2%) received at least one critical prehospital intervention. Upper airway suction was performed in 39 cases (1.0%), endotracheal intubation (all causes) in 36 cases (0.9%), and intraosseous access in 21 cases (0.5%). General anaesthesia was induced in 29 cases (0.7%). Seventeen patients (0.4%) received cardiopulmonary resuscitation and two patients received manual defibrillation (< 0.1%). 3,278 patients were admitted to the hospital and assigned a diagnosis when discharged. The most common diagnoses were assigned within the International Statistical Classification of Diseases and Related Health Problems 10th Revision Chapter XVIII (Symptoms, signs and abnormal clinical and laboratory findings, not elsewhere classified), which includes febrile convulsions. 1,437 patients (43.8%) were assigned diagnoses within this diagnosis group.

The overall 7-day mortality in the cohort was 0.74%, 30-day mortality was 0.82%, and 90-day mortality was 1.02%.

**Conclusion:**

Prehospital critical interventions are rarely performed in children under the age of 7 years. The low frequency of these interventions may have implications for maintaining the clinical routine of the prehospital care providers.

## Introduction

Prehospital care of acutely ill or injured children can be described as a field with high risk and low tolerance for mistakes. Children, and in particular, the youngest children, differ from adults anatomically, physiologically, and emotionally [1]. Children and infants are frequent patients in the prehospital setting [2] and during the past decade, prehospital research on overall paediatric issues has been given more attention [3–5]. Although implicitly, the acute treatment of a teenager differs considerably from the acute treatment of a toddler or infant, most prehospital studies in children have broadly defined children as being below 16 or 18 years of age [2–7], leaving us with little knowledge of the treatment of the very young children.

Although life-threatening events are infrequent among children [6, 8–10], the treatment of children with severe illness or injury may be stressful for healthcare professionals [11–14]. Emergency medical system (EMS) providers may have critical knowledge gaps in paediatric care, including paediatric airway management, general paediatric skills, and also responder anxiety concerning paediatric care [15]. Even in a prehospital system comprising anaesthesiologists or other physicians, the capability to perform critical interventions in children may differ as not all physicians delivering prehospital care are routinely tasked with treating critically ill children. In Denmark, the most advanced tier in the prehospital system consists of a ground-based anaesthesiologist-manned mobile emergency care unit (MECU) or an anaesthesiologist-manned helicopter emergency medical service (HEMS). One, or both of these, are usually dispatched along with an ambulance when the EMS responds to calls concerning critically ill children [16]. The Danish EMS employs anaesthesiologists from all parts of in-hospital anaesthesia departments. Thus, the anaesthesiologists on call may have different experiences and training levels regarding intubation, vascular access, intraosseous access, and advanced life support in critically ill or injured children and infants. These interventions may be difficult to perform in children and infants without the required training and experience [17].

The ability to respond adequately to the needs of an acutely ill or injured child has been associated with decreased morbidity and mortality after emergency treatment [18, 19]. The concept of ‘paediatric readiness’ hence comprises continuous education of personnel, standardised choice of age- and weight-specific equipment and allocation of competent staff to adequate settings [18, 19]. Repetition of practical skills among operators is likely to improve patient outcomes and may increase operator resilience in stressful situations [20].

Knowledge of the extent of prehospital critical interventions may therefore inform future educational initiatives in prehospital services. While other studies have applied other age levels, defining a patient as a child up to 16 or 18 years [2–7, 21], our study focuses on the extent of life-saving critical prehospital interventions performed in small children. The primary outcome was thus critical interventions (defined as endotracheal intubation, intraosseous access, upper airway suction, prehospital anaesthesia, cardio-pulmonary resuscitation, or defibrillation) in children below the age of 7 years.

The secondary outcome was the overall pattern of morbidity and mortality (7-day, 30-day and 90-day) in children below the age of 7 years who were treated by the MECU unit based in Odense, Denmark.

## Methods

### System setting

The Danish EMS is a tax-funded three-tiered system. The first tier is an ambulance manned by two emergency medical technicians, one of which typically is a paramedic. The second tier consists of one paramedic in a rapid-response car. The third tier consists of an anaesthesiologist assisted by an emergency medical technician or paramedic in either a ground-based unit (MECU) or a helicopter-based unit [16]. At the beginning of the observation period, the MECU in Odense, Denmark, serviced a mixed urban/rural population of 250,000 to 490.000 people. In the last ten years, due to a more constant prehospital structure in the region with six MECUs scattered in the health region, the population covered by the MECU in Odense has been constant at around 260.000 people. Approximately 68% of the covered population is living within the city of Odense, the third largest city in Denmark. The MECU supplements the ambulance service 24/7/365 and responds to approximately ten calls daily, corresponding to 26% of all emergency calls dispatched as priority 1 [22]. In all urgent cases in children (defined as missions dispatched as priority 1), the MECU is dispatched along with an ambulance.

### Data collection

We retrospectively reviewed the medical records from the MECU in Odense from October 1, 2007, to December 31, 2020. Data routinely collected in the medical records include the unique Danish Civil Personal Register number, patient age at the time of contact, injury/illness severity according to the National Advisory Committee for Aeronautics (NACA) score [23], and critical interventions performed in the patients. We retrieved data regarding the length of stay at the hospital and the diagnosis from the Danish National Patient Registry [24]. We further retrieved data concerning migration status and 7-day, 30-day and 90-day mortality from the Danish Civil Personal Registry [25].

### Definition of critical interventions

We defined “life-saving critical interventions” as intraosseous (IO) access, endotracheal intubation, upper airway suction, anaesthesia, cardiopulmonary resuscitation, and defibrillation.

Data on intravenous (IV) access was collected. However, the procedure was not defined as a critical intervention, since IV access in some cases is placed without presence use for further treatment.

### Inclusion and Exclusion criteria

Inclusion:

- Children below 7 years treated by MECU, Odense, Denmark

Exclusion:

- Cancelled missions, standby missions, reprioritised missions, and other missions without patient contact.
- Missions with unidentified patients.
- Duplicate missions.

### Data management and handling

Data were collected from October 2007 through December 2020. If a patient had more than one contact with the MECU during the observation period, each contact was included.

The cases were divided into two groups. Group 1 received critical interventions performed by the prehospital anaesthesiologist, while Group 2 did not receive critical interventions.

### Statistical analyses

We used the Pearson Chi-square test to compare the group who received interventions (Group 1) and the group who did not receive interventions (Group 2) regarding sex and age group. The groups’ NACA scores were compared using Fisher’s exact test with Monte Carlo simulation. Wilcoxon rank-sum was used to compare age as a continuous variable. Pearson Chi-square test was used to compare transport/on-scene time, admission length, and 7-, 30-, and 90-day mortality rates, respectively. A predetermined *p*-value of < 0.05 was accepted as significant.

StataMP 18.0 (StataCorp, College Station, Texas, USA) was used for all analyses except Fisher’s exact test with Monte Carlo simulation, conducted in RStudio with R version 4.3.2.

### Ethical approvals

The study was performed in accordance with all relevant national guidelines and regulations. The Regional Judicial Office of the Region of Southern Denmark (Ref. no. 23/8531) and the Judicial Office of Odense University Hospital (Ref no. 22/54631) approved the project. According to the Act on Processing of Personal Data, in register-based studies approved by the Danish Patient Safety Authorities, no consent is required to use data already entered into the registry. Thus, no further approvals are necessary according to Danish law (26]. In addition to the required approvals, all data handling was carried out in accordance with Danish and European legislation concerning person-identifiable data [26, 27].

## Results

From October 2007 through December 2020, the anaesthesiologist-manned MECU was dispatched to 54,482 missions. 9,722 (17.8%) missions were cancelled before the arrival of the MECU. 422 (0.8%) missions were stand-by missions without any patient contact. 1,118 (2.1%) missions were reprioritised.

**Fig. 1:**
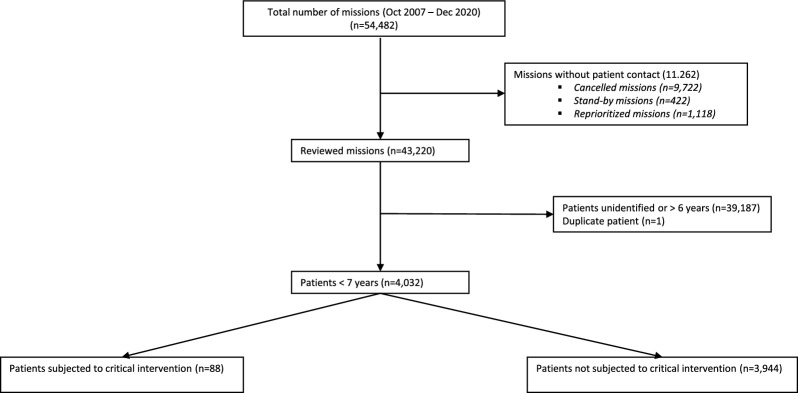
Flowchart

The remaining 43,220 missions were reviewed. In 4,037 (9.3%) of the missions, the MECU treated children below the age of seven years. One mission with duplicate registration and four unidentified patients were excluded. The total number of included missions was thus 4,032, involving 3,276 unique children below the age of seven at the time of the mission (See Fig. 1). There was an even distribution between sexes in children below the age of one year. In older children, male sex dominated the groups (p < 0.001, data not shown). For demographics, NACA score, response time, on-scene time, and transport time to hospital, see Table 1.

**Table 1:** Demographics of the study population, National Advisory Committee for Aeronautics score of the severity in cases of medical emergencies, and time expenditure (Response time, on-scene time, transport time to hospital)

		Total	Children subjected to critical interventions	Children not subjected to critical interventions	*p*-value
		4,032	88	3,944	
Sex, n (%)	Boys	2,360 (58.5%)	216 (61.9%)	2,144 (58.2%)	0.91
	Girls	1,672 (41.5%)	133 (38.1%)	1,539 (41.8%)	
Age	Years, median (IQR)	1.6 (0.9–3.1)	1.4 (0.4–2.2)	1.6 (0.9–3.0)	0.009
Age Group, n (%)	0–2 months	283 (7.0%)	14 (15.9%)	269 (6.8%)	0.006
	2–12 months	854 (21.2%)	20 (22.7%)	834 (21.1%)	
	12–24 months	1,249 (31.0%)	27 (30.7%)	1,222 (31.0%)	
	> 24 months	1,646 (40.8%)	27 (30.7%)	1,619 (41.0%)	
NACA score, n (%)	0	216 (5.4%)	1 (1.1%)	215 (5.8%)	< 0.001
	1	489 (12.1%)	7 (8.0%)	482 (12.2%)	
	2	189 (4.7%)	0 (0.0%)	189 (4.8%)	
	3	1,464 (36.3%)	12 (13.6%)	1,452 (36.8%)	
	4	361 (9.0%)	8 (9.1%)	353 (9.0%)	
	5	50 (1.2%)	14 (15.9%)	36 (0.9%)	
	6	25 (0.6%)	21 (23.9%)	< 5 (< 0.1%)	
	7	13 (0.3%)	5 (5.7%)	8 (0.2%)	
	Not registered	1,225 (30.4%)	20 (11.7%)	1,205 (30.6%)	
Response time	N	3,976 patients*	88 patients	3,888 patients	
	Minutes, median (IQR)		7.0 (5.0;11.0)	8.0 (5.0;11.0)	0.32
On-scene time	N	3,985 patients*	88 patients	3,897 patients	
	Minutes, median (IQR)		13.0 (7.0;27.0)	11.0 (8.0;15.0)	< 0.013
Transport time to hospital	N	813 patients*	57 patients	756 patients	
	Minutes, median (IQR)		13.0 (8.0;17.0)	11.0 (7.0;17.0)	0.58

*Number of patients with available data

*Patients where times were registered

Stratified according to prehospital critical intervention or not. Sub groups containing 4 or fewer patients are noted < 5 patients according to Danish regulations concerning presentation of register-based data [46]

663 patients (16.4%) were treated and released at the scene. 88 (2.2%) patients received at least one critical intervention (Group 1), while 3.944 (97.8%) were not subjected to critical interventions (Group 2). Within group 1, 47 children received IV access. Within group 2, 261 children received IV access.

The life-saving prehospital critical interventions registered were intraosseous access (n = 21), endotracheal intubation (n = 36), upper airway suction (n = 39), prehospital anaesthesia (n = 29), cardio-pulmonary resuscitation (n = 17), and defibrillation (n = 2) (Table 2).

**Table 2:** Procedures and critical interventions carried out stratified according to age group

Procedures	N (total population for age group)	Proportion of total population in the age group subjected to critical intervention (%)
IO access	21 (4,032)	0.5
0–1 years	11 (1,137)	1.0
1–2 years	5 (1,249)	0.4
2–6 years	5 (1,646)	0.3
Upper airway suction	39 (4,032)	1.0
0–1 years	17 (1,137)	1.5
1–2 years	11 (1,249)	0.9
2–6 years	11 (1,646)	0.7
Endotracheal intubation	36 (4,032)	0.9
0–1 years	14 (1,137)	1.2
1–2 years	12 (1,249)	1.0
2–6 years	10 (1,646)	0.6
CPR	17 (4,032)	0.4
0–1 years	7 (1,137)	0.6
1–2 years	8 (1,249)	0.6
2–6 years	< 5 (1,646)	< 0.3
Defibrillation	< 5 (4,032)	< 0.1
0–1 years	< 5 (1,137)	< 0.4
1–2 years	< 5 (1,249)	< 0.4
2–6 years	0 (1,646)	0
Anaesthesia	29 (4,032)	0.7
0–1 years	7 (1,137)	0.6
1–2 years	8 (1,249)	0.6
2–6 years	14 (1,646)	0.9

Total number of patients is given in parentheses. Sub groups containing 4 or fewer patients are noted < 5 patients according to Danish regulations concerning presentation of register-based data [46]

Critical interventions were more often carried out when the patient was under two years of age (Table 2).

A NACA score [23, 28, 29] was registered in 2,807 cases with the majority of cases (58.5%) given a NACA score of 3 or less. The NACA score differed between groups. Higher NACA scores were more frequent among children subjected to critical interventions. (See Table 1). The median MECU on-scene time in Group 1 was significantly longer than on-scene time in Group 2, 13 min (Quartiles 7.0–27.0) and 11 min (Quartiles 8.0–15.0), respectively (*p* < 0.013). The transport time to and from the scene to the hospital was similar in both groups (Table 1.) 841 (20.9%) of the patients were escorted to the hospital by the anaesthesiologist. Of the children receiving a critical intervention in the prehospital period, 77 patients were admitted to a hospital. Of these, 20.8% (16 patients) were admitted to the hospital for a period longer than 72 h, compared to the non-intervention group, where 5.3%% (170/3,201 patients) were admitted for longer than 72 h (*p* < 0.001). In both groups, a length of stay at the hospital of less than 24 h was most frequent, 58.4% and 82.9%, respectively. Overall, 41 children (1.02%) died within 90 days of MECU contact. The 7-day, 30-day and 90-day mortality was significantly higher in Group 1 (*p* < 0.001) (Table 3). In 17 cases, the child was declared dead at the scene (0.4%).

**Table 3:** Admission length and mortality rates distributed according to critical intervention carried out (Group 1) and no critical intervention carried out (Group 2)

Hospital-admitted patients (N = 3,278)	Children subjected to critical intervention (Group 1)	Children not subjected to critical intervention (Group 2)	*p*-value
Admission length	N = 77	N = 3,201	< 0.001
< 24 h	45 (58.4%)	2,653 (82.9%)	
24–72 h	16 (20.8%)	378 (11.8%)	
> 72 h	16 (20.8%)	170 (5.3%)	
*Mortality*			
Death within 7 days	18 (20.5%)	12 (0.3%)	< 0.001
Death within 30 days	20 (22.7%)	13 (0.3%)	< 0.001
Death within 90 days	21 (21.9%)	20 (0.5%)	< 0.001

In 3,278 children, an in-hospital diagnosis was registered. Of 663 patients released at the scene and a further 91 patients admitted to a hospital, no in-hospital diagnosis was registered.

The most frequently (43.8%) assigned diagnoses were within the ICD-10 Chapter XVllI (*Symptoms, signs, and abnormal clinical and laboratory findings, not elsewhere classified*), which includes febrile convulsions/seizures. Of the 1,437 children assigned a discharge diagnosis within this chapter, 1,287 (89.6%) were diagnosed with febrile convulsions The second most frequently (16.4%) assigned diagnosis was registered within the ICD-10 chapter X *(Diseases of the respiratory system)*. The ICD-10 Chapter XIX (*Injury, poisoning and certain other consequences of external causes*) includes all injuries, poisoning, and allergies and constituted the third most frequently assigned diagnosis chapter (14.6%).

The hospital discharge diagnoses assigned within the ICD-10 chapter classification [30] are shown in Table 4.

**Table 4:** Hospital discharge diagnosis within ICD-10 chapters

ICD-10 chapter	Diagnosis group		Children subjected to critical intervention (Group 1)	Children not subjected to critical intervention (Group 2)
			N = 77 (100.0%)	N = 3,201 (100.0%)
Chapter I	Certain infectious and parasitic diseases	(A00-B99)	7 (9.1%)	106 (3.1%)
Chapter III	Diseases of the blood and blood-forming organs and certain disorders involving the immune mechanism	(D50-D89)	0	< 5
Chapter IV	Endocrine, nutritional and metabolic diseases	(E00-E90)	0	38 (1.2%)
Chapter V	Mental and behavioural disorders	(F00-F99)	0	17 (0.5%)
Chapter VI	Diseases of the nervous system	(G00-G99)	10 (13.0%)	135 (4.2%)
Chapter VII	Diseases of the eye and adnexa	(H00-H59)	0	< 5
Chapter VIII	Diseases of the ear and mastoid process	(H60-H95)	0	9 (0.3%)
Chapter IX	Diseases of the circulatory system	(I00-I99)	10 (13.0%)	12 (0.4%)
Chapter X	Diseases of the respiratory system	(J00-J99)	7 (9.1%)	530 (16.6%)
Chapter XI	Diseases of the digestive system	(K00-K93)	< 5	22 (0.7%)
Chapter XII	Diseases of the skin and subcutaneous tissue	(L00-L99)	0	12 (0.4%)
Chapter XIII	Diseases of the musculoskeletal system and connective tissue	(M00-M99)	0	< 5
Chapter XIV	Diseases of the genitourinary system	(N00-N99)	0	9 (0.3%)
Chapter XVI	Certain conditions originating in the perinatal period	(P00-P96)	0	66 (2.1%)
Chapter XVII	Congenital malformations, deformations and chromosomal abnormalities	(Q00-Q99)	0	15 (0.5%)
Chapter XVIII	Symptoms, signs and abnormal clinical and laboratory findings, not elsewhere classified	(R00-R99)	30 (39.0%)	1,407 (44.0%)
Chapter XIX	Injury, poisoning and certain other consequences of external causes	(S00-T98)	10 (13.0%)	470 (14.7%)
Chapter XXI	Factors influencing health status and contact with health services	(Z00-Z99)	3 (3.9%)	341 (10.7%)

Patients stratified according to critical intervention carried out (Group 1) and no critical intervention carried out (Group 2). Sub groups containing 4 or fewer patients are noted < 5 patients according to Danish regulations concerning presentation of register-based data [46]

## Discussion

Only 2.2% of children below the age of seven years whom the MECU treated required advanced life-saving prehospital interventions. By far, the most common diagnosis in children treated by the MECU in Odense was febrile convulsions. This is in concordance with other studies stating that febrile convulsions are frequently occurring in the paediatric prehospital population [31]. Febrile convulsions very seldom require treatment beyond rectal or mucosal administration of benzodiazepines but are usually self-limiting and without the need for other interventions [32]. In a population of 333 children with febrile convulsions, only 6% of the cases were treated with intravenous diazepam in the prehospital setting [33]. When critical interventions were performed prehospital, on-scene time was significantly longer, with notably greater variation in the interquartile range from 13 to 27 min. This could indicate that performing procedures is time-consuming and delays arrival at the hospital. Therefore, in some instances, a load-and-go approach may have been instituted and the MECU anaesthesiologist could have chosen not to perform a critical intervention to minimise the time before arrival at the hospital. Our study is carried out in an area with relatively short distances and transport times from scene to hospital are generally brief. A nationwide Danish study reported critical interventions performed in 20% of the children treated by the helicopter emergency medical service (HEMS) [34] and a recent study carried out in Norway, report that of the included 309 children who were treated by a HEMS physician, 42% received critical interventions [35]. Both studies were carried out in larger areas with longer distances and transportation by helicopter. Due to NACA scores, the severity of the included patients’ injury/illness, was similar.

A NACA score was available in 70% of all missions. Incomplete prehospital registration of vital signs is often associated with non-urgent cases [36]. In our study, we assume that the missions where NACA score was not registered were cases where the score would have corresponded to NACA 2 or less. Based on the prehospital NACA scores, over 50% of the children that the MECU services, do not require acute interventions. This is in concordance with other recent studies [35, 37] and could suggest that the barrier for dispatching the MECU is low regarding children. Our data show that when the MECU performed critical interventions, the child was severely ill or injured and was often admitted to the hospital for more than 72 h. When the NACA score was above 3, critical interventions were more often performed than not. Seventeen patients were declared dead at the scene. Most of these patients were described as dead at the arrival of the MECU. In seven of the patients, no critical interventions were thus performed prehospital.

The 90-day mortality rate of all included cases was 1.02%. In Denmark, the yearly mortality rate in children below seven years is 0.12% [38]. 7-day, 30-day and 90-day mortality was significantly higher among the children where the MECU performed critical interventions. In children without any critical prehospital interventions, the mortality also exceeded the mortality in the background population.

### Interpretation and relation to other studies

Several studies show that prompt access to appropriately trained medical care providers and the necessary equipment reduces the mortality rate in acutely ill children [39–41]. As we have shown, prehospital anaesthesiologists rarely perform intubation or other advanced procedures on small children in the prehospital setting. Endotracheal intubation is an essential skill for airway management and a key skill for an anaesthesiologist. However, Maek et al. [38] found that children up to six years old were less likely to be intubated in the prehospital setting compared to adults, even though an indication for intubation was established. Brownstein et al. [21] studied 355 children below 15 years, with a median age of 3 years. The prehospital intubations were performed by paramedics. The study showed that not only was intubation less often attempted in critically injured children, but the complication rate in intubated children was 22.6%, while the number of failed intubation attempts could not be determined. All Danish MECU anaesthesiologists are trained in intubation and daily perform intubations in adults at their primary employment at the hospital. Only a few prehospital anaesthesiologists working in the prehospital field have specific paediatric training and exposure to sick children daily. Our study did not measure skill proficiency, such as the success rate of a given procedure or the number of attempts required to place the tracheal tube. Two recent studies, however, explored the success rate of endotracheal intubation in children in the prehospital setting [42, 43]. AlGhamdi et al. completed a meta-analysis of 38 studies, with the majority of the included studies performed with a retrospective design [42]. The upper age limits of the patients were 12–18 years and paramedics often performed the intubations. The overall success rate was 82.5%, with a first-pass success rate of 77.2%. Analysing data from the Finnish HEMS manned with anaesthesiologists, Elonheimo et al. [43] demonstrated that the overall success rate performing endotracheal intubation in children within the age of 15 years by a HEMS physician is 100% with 86% of the intubations carried out at first attempt. However, the first-pass success rate was significantly lower when the child was below one year old. Assuming that there is some overlap in procedural skills between adults and children, frequently performing procedures in adults may help maintain the same skills in the paediatric population. However, given the anatomic and physiologic differences between children and adults, presumptions about translating the proficiency of adult procedures to those of children must be approached cautiously, especially regarding the youngest children.

The unplanned critically ill child´s first encounter with the health care system usually takes place outside the hospital. The educational level of most prehospital Danish physicians is that of board-certified specialists in anaesthesiology and there are no formal requirements for specific training or routine in paediatric care. However, in Denmark, elective surgical interventions in children are planned in advance. Within Odense University Hospital, children below three years in need of anaesthesia are usually treated by a specialised paediatric anaesthesiologist. Furthermore, children up to 20 kg undergoing surgery are anaesthetised by an experienced anaesthesiologist. The upper limit of seven years in our investigated population corresponds to a mean patient weight of 20 kg [44]. By extension, we have thus sought to evaluate the treatment of the children who would have been treated by specialised paediatric anaesthesiologists or at least very experienced anaesthesiologists in the in-hospital setting.

IV access does not constitute a critical intervention since one could place an IV access only due to convenience or to spare subsequent colleagues the work if the child is admitted. Nevertheless, the pain experienced by the child during this procedure [45] in combination with the physicians´ potential lack of routine could influence the decision-making concerning placing one.

Our study is based on data from a period of 159 months. In that period a total of 308 intravenous access was successfully placed. This equals less than two intravenous accesses in small children per month in the whole unit. In general, attempts at inserting an intravenous access often precede attempts of inserting an intraosseous access. However, we found that intraosseous access was significantly more frequent when the patient was younger than two years. This could suggest that intravenous access is associated with a lower success rate when the patient is younger than two years. Furthermore, the proportion of children having intravenous access inserted and the proportion of children having intraosseous access placed suggest that treating and performing critical interventions in the youngest children could be associated with more anxiety and ensuing hesitation considering a commitment to perform the procedure within the caregiver. Mockler et al. [20] investigated children aged up to 10 years in a German helicopter. The research group concluded that on-the-job training and mission experience alone are insufficient for acquiring and maintaining the competencies needed to care for critically ill or injured children. Our results may be interpreted as supporting that notion.

### Strengths and limitations

Strengths of the study include the large number of patients registered during more than 13 years and the low number of patients lost to follow-up. The study was performed retrospectively, and limitations caused by information bias may be present. Information in the Database MECU Odense was registered when, or shortly after, treating the patient. However, the MECU could be dispatched to another mission before completing data entry from the previous task, resulting in recall bias.

## Conclusion

Prehospital critical interventions in children below the age of seven years are rarely performed. The low frequency of these interventions may have implications for maintaining a performance routine for the prehospital anaesthesiologists. Prehospital paediatric readiness may not be maintained in the EMS. Developing strategies to maintain paediatric technical skills could increase paediatric readiness and ensure effective care for critically ill or injured children.

## Data Availability

Within the limits of the Danish legislation, anonymised data are available from the corresponding author on reasonable request.

## Abbreviations

EMS: Emergency medical system
ICD-10: International statistical classification of diseases and related health problems 10th revision
IO: Intraosseous
IV: Intravenous
MECU: Mobile emergency care unit
NACA: National advisory committee for aeronautics
OR: Odds-ratio
